# Medical-Radiation-Shielding Film Fabricated by Imitating the Layered Structure Pattern of Abalone Shell and Verification of Its Shielding Effect

**DOI:** 10.3390/ma16247700

**Published:** 2023-12-18

**Authors:** Seon-Chil Kim

**Affiliations:** 1 Department of Medical Informatics, Keimyung University, 1095 Dalgubeol-daero, Daegu 42601, Republic of Korea; chil@kmu.ac.kr; 2 Department of Biomedical Engineering, Keimyung University, 1095 Dalgubeol-daero, Daegu 42601, Republic of Korea

**Keywords:** medical radiation, lead, shielding, shielding sheet, tungsten

## Abstract

Radiation-shielding clothing for medical workers must be light and thin, thus ensuring flexibility. However, controlling the thickness and weight is limited by shielding performance requirements. This study aims to improve shielding performance by considering a shielding structure that mimics the internal structure of an abalone shell. Two shields were produced: a sheet made with a carrier process using a liquid polymer and tungsten mixture, and a fillet made by compounding the same material and laminated using a heat-treatment press after the injection process. The tungsten content and thickness were the same at 85 wt% and 0.3 mm, respectively. In the high-energy region, the shielding film based on the laminated structure of abalone shells showed a shielding rate that was higher by more than 7%. Compared to that of a 0.3 mm lead plate, the shielding ratio of the shielding film was approximately 16% lower at 120 kVp, thereby confirming the radiation-shielding effect of the layered-structure shielding film. Therefore, it is concluded that the laminated structure of the shielding film, which is identical to the internal laminated structure of the abalone shell, expands the impact area of incident radiation and attenuates the energy intensity, thereby improving the medical-radiation-shielding performance.

## 1. Introduction

Radiation shielding in medical institutions has traditionally relied on lead owing to its softness, ease of processing, and cost-effectiveness [1]. Heat treatment is the most commonly used method for processing lead into a radiation-shielding material, and it involves mixing lead rubber powders [2]. The shielding performance can be adjusted by varying the mixing ratio of lead, and the process technology is relatively simple [3]. Lead has been widely used in medical facilities for equipment and facility shielding because of its competitive price. However, lead is a heavy metal and poses risks of lead poisoning to the human body [4]. Therefore, there has been a recent trend to shift toward environmentally friendly shielding materials such as tungsten, bismuth oxide, and barium sulfate [5]. While eco-friendly materials may not always provide the same level of shielding performance and cost-effectiveness as lead, they can offer superior shielding performance in some cases. Particularly, X-ray radiation is commonly used in medical applications. While manufacturing shielding tools for radiation reduction, there are advantages in being able to uniformly adjust the thickness at a certain distance. For example, in the case of aprons, which are shielding garments, the shielding standard is typically set to the thickness of lead equivalent, such as 0.25 mmPb, 0.30 mmPb, and 0.50 mmPb, depending on the desired defense level and dose–distance considerations [6]. However, inside medical devices, tin, copper, aluminum, and other alternatives to lead are often used to form shielding walls. Achieving an equivalent shielding performance to that of lead with these materials requires increased material mixing and density of the shielding body, resulting in thicker shielding walls when compared to lead with the same thickness.

Tungsten is recognized as an environmentally friendly alternative to lead for radiation shielding because of its comparable performance, making it an excellent choice for lightweight applications in devices, machinery, and clothing [7]. With an atomic number of 74, an atomic weight of 183.84 g/mol, and a density of 19.25 g/cm^3^, tungsten’s shielding capabilities rival those of lead. However, its high melting point of 3400 °C presents challenges for direct processing [8]. Consequently, it is often utilized in sheet form by combining it with a polymer in powder form to facilitate processing [9]. Nevertheless, this processing method may compromise shielding performance as tungsten content decreases in the same area and thickness, resulting in a lower density of the fabricated shield and directly impacting its effectiveness. Addressing this issue requires careful consideration of the internal structure of the shielding body, as the attenuation of incident radiation relies on interactions during the transmission and absorption of radiation [10].

In this study, our goal is to enhance shielding performance by increasing the density within the shield while creating a patterned structure that promotes interactions with incident radiation. To achieve this, we draw inspiration from the layered structure of the abalone shell and aim to implement a radiation-shielding film with a similar structure [11]. We manufactured a multi-layered shielding film through heat treatment, utilizing injection-molded tungsten films of the same size, and then assessed changes in shielding performance.

Currently, numerous studies are exploring the development of more effective engineering models by drawing inspiration from human or animal biomimicry [12]. Examples include non-slip pads based on the foot sole pattern of baboon lizards, swimming suits incorporating shark scales, and adhesive models mimicking octopus suckers, providing valuable insights for engineering approaches [13,14]. Of particular interest is the abalone shell, which undergoes size changes as it grows from the inside to the outside over an extended period [15]. The hexagonal aragonite in the shell is arranged in a zigzag shape to prepare for external impacts, forming a layered structure with 10 nano-protein layers [16].

This unique structural pattern is believed to play a role in attenuating the intensity of incident radiation energy [17]. While previous studies have investigated the laminated structure of abalone shells in terms of fracture toughness, compressive strength, bending strength, and tensile strength, this study is the first to explore a method of inducing interaction by expanding the collision area of particles, such as radiation [18]. In this study, we employed an injection process using tungsten and polymer resin to replicate the same pattern as the abalone shell, and the resulting shielding film was molded to achieve a certain level of strength [19]. However, we further developed a process technology that involves repeated compression after injection to create a multi-layered laminated structure similar to that of the abalone shell. The shielding performance of the laminated shielding film was verified by comparing it with a shielding sheet and a lead plate of the same thickness in this experiment [20]. The results confirmed that the layered structure of the shielding film has the potential to improve the overall shielding performance.

## 2. Methods

X-rays, which are commonly used in medical radiation, follow the exponential attenuation law (Beer Lambert’s law) based on the thickness of the shielding film, as shown in Equation (1) [21]:

(1) $$\frac{I}{I_{0}}=e^{-\mu \chi },$$

where $I_{0}$ and I represent the intensities of the incident and transmitted beams, respectively, χ denotes the thickness of the shield, and μ is the linear attenuation coefficient. The mass decay coefficient ($\mu_{m}$) is used to express the attenuation of the coefficient when passing through the interior of the shielding film, which explains the probability of interaction between the shielding material and the photons, as shown in Equation (2) [22].

(2) $$\mu_{m}=\frac{\mu }{\rho }=-\frac{1}{\rho \chi }ln\left(\frac{I}{I_{0}}\right)=\frac{1}{\rho N}\frac{dN}{d\chi }.$$

The probability of interaction increases with the number of particles while passing through the thickness of the shielding film. Therefore, if continuous attenuation is desired, a thickness of dχ can be created. In the case of a layered-structure density ρ where the same thickness is repeated, the shielding effect $S_{i}$ can be calculated according to the total impact cross-sectional area $R_{S}$, continuously affected by the density $\rho_{i}$, as shown in Equation (3) [23]. Consequently, the laminated internal structure of the shielding film can be understood as a continuous collision cross-section that enhances the probability of interaction for incident radiation particles [24].

(3) $$\sum_{Rs}=\sum_{i}\rho_{i}\left(\Sigma_{\frac{Rs}{\rho }}\right)i.$$

**Figure 1:**
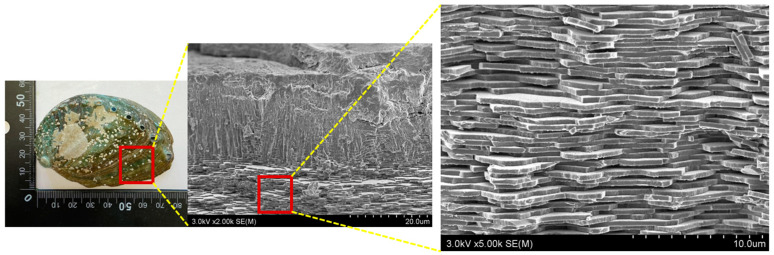
Magnified electron microscope image of the outer and inner layers of the abalone shell. An enlarged image of the appearance and cross-section of abalone skin.

First, the structural form of the abalone shell was visually analyzed using an electron microscope. As depicted in Figure 1, the outer layer of the abalone shell exhibits a vertical columnar structure, whereas the inner layer shows a stacked horizontal structure. This dual-layered structure is well suited for dispersing external pressure, as noted in previous studies [25]. In this study, the inner layer of the abalone shell, which consists of multiple laminated structures, is the specific focus for shielding purposes. The laminated structure of the inner layer allows for an active response to external shocks, making it an ideal candidate for shielding. By creating a structure that allows incident radiation to repeatedly penetrate multiple protein layers and air gaps between them, the cross-sectional area of collision can be significantly increased. Such a structure has the potential to enhance the probability of interaction between incident radiation and the shielding material [26].

For this experiment, tungsten (Tungsten, W, 98.9%, NanGong XinDun Alloys Spraying Co., Ltd., Nangong, China) with an average particle size of 4.28 ± 0.1 μm was selected as the shielding material. For particle analysis, a nanofiber mat (WN40) was cut into 50 mm. This was fixed in a 3 cm^2^ circular holder and analyzed without any additional preprocessing.

**Figure 2:**
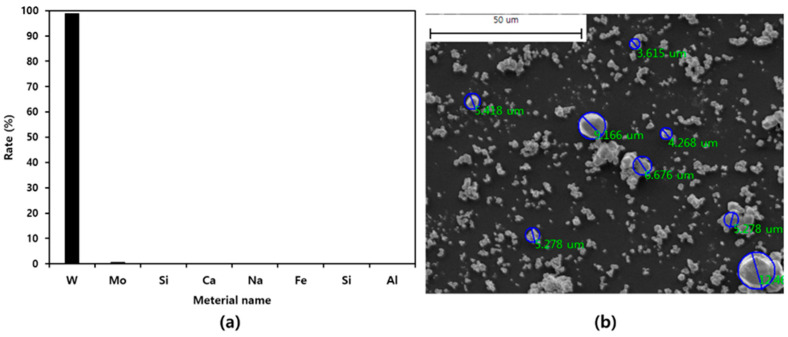
Tungsten powder properties: (**a**) XRF, (**b**) SEM. Component analysis and particle size analysis photo of tungsten material used as shielding material.

The XRF and SEM results of tungsten powder are shown in Figure 2. Eight elements were detected through XRF analysis, with tungsten, molybdenum, and silicon composing 98.91%, 0.68%, and 0.13% of the material, respectively. In addition, it was confirmed that the powder contained less than 0.1% of other elements, making it mostly tungsten. The sizes of 134 tungsten particles were confirmed using SEM images, and the largest particle was measured at 12.636 μm. The measured particles were measured to have an average size of 3.946 μm, and it was confirmed that most particles were 4.0 μm or less. An additional material used to create a structure that mimics the inner layer of an abalone shell is a polymer resin. Polyamide nylon resin (Polyamide, P-21, Mw = 100,000–150,000, Songwon, Ulsan, Republic of Korea) was used as the polymer resin.

**Figure 3:**
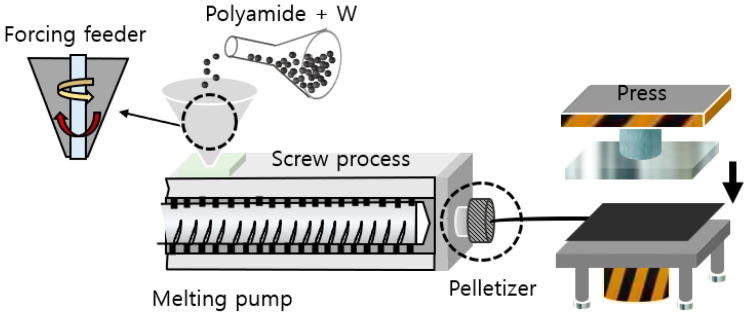
Press forming process to create laminated structure. Illustration depicting the process for producing a shielding film.

To replicate the shielding performance in the composite material, the tungsten content was set at 85 wt% [27]. Additionally, to assess the improvement in shielding performance during the experiment, a single-structured sheet was manufactured by blending the same materials and employing the calendering process. A lead plate (Pb, 99.5%, Seung Han, Naju-shi, Republic of Korea) was used as a benchmark for an absolute evaluation of shielding performance. First, by mixing tungsten powder and resin, the shielding film manufacturing process produced a fillet that could be used in an injection molding machine [28]. The resulting shielding film repeatedly reproduced the laminated structure through press rolling, as shown in Figure 3. Here, the laminated structure was affected by the internal capacity of the injection machine and the press mold, and a method of lowering the initial capacity to about 50 cc and then increasing it was applied in order to increase the capacity of tungsten-based compound materials. The speed of the injection machine was 350 rpm, the injection pressure was 65–70 kg/m^2^, and the holding pressure was 75 kg/m^2^. The temperature was increased from 80 °C to 150 °C to control the solidification rate to form a laminated structure. The thermal cylinder pressure press was set to a maximum value of 250 kg/cm^2^, and gauge pressure was added to the area of the shield at a temperature of 180–220 °C. The shielding film, manufactured in a mold, had a final thickness of 0.3 mm and horizontal and vertical lengths of 500 mm.

**Figure 4:**
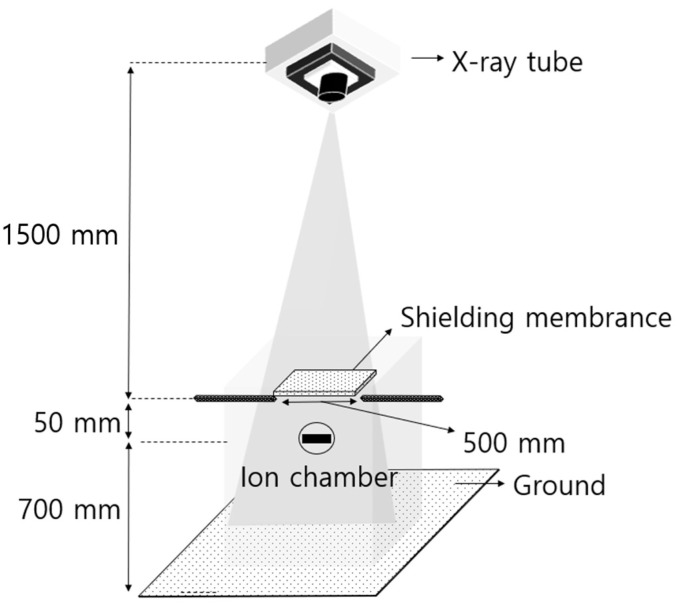
Shielding performance evaluation method. X-ray generator and shielding body, schematic diagram to explain the location of the measurement sensor and prevent backscattering lines.

The shielding sheets made for the comparison experiments also shared the same tungsten content of 85 wt%, and the mixture of polymer resin and tungsten powder was processed to a thickness of 0.3 mm using the Lawler rolling process [29]. The manufactured shielding film and shielding sheet were then analyzed using SEM for tungsten, a single shielding material. The prepared sample was sectioned into thin films using a microtome (Leica, Wetzlar, Germany, RM2235) and observed using an electron microscope (field-emission scanning electron microscope, Hitachi, Tokyo, Japan, S-4800). Each cross-section was observed at the same magnification to visually compare and analyze the degree of dispersion of the shielding material in the internal structure [30]. To evaluate the shielding performance, a diagnostic X-ray generator (Toshiba E7239, 150 kV-500 mA, 1999, Tokyo, Japan) was used with a fixed tube current of 200 mA, which falls within the range of medical radiation exposure. The experiment was conducted using a consistent tube current of 200 mA. The procedure was performed three times across different tube voltages, namely, 40 kVp, 60 kVp, 80 kVp, 100 kVp, and 120 kVp, which are commonly used in human body radiography. The results reported are the average values derived from these three trials. The radiation dose detector was used after verification and calibration with the Radical Corporation Mo.9517 Radiation Monitor, Mo10×5–6, 6 cc Ion chamber (Radical Corp., Santiago de Surco, Peru). The experimental method for evaluating radiation-shielding performance followed the lead-equivalent test method for X-ray protective products (KS A 4025: 1990, confirmed in 2009) in the Korean Industrial Standard, as shown in Figure 4 [31].

## 3. Results

**Figure 5:**
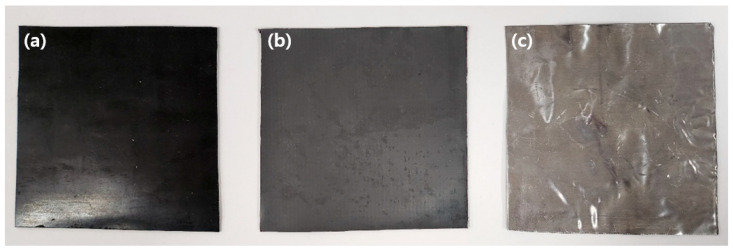
Shape of the shield manufactured as a prototype. (**a**) Shielding film made in a laminated structure by adding a molding compression process; (**b**) shielding sheet manufactured using the calendering process; (**c**) lead plate.

A shielding film was produced mimicking the structure of an abalone shell and its inner layer, while a shielding sheet of the kind currently used in medical institutions was also manufactured. As shown in Figure 5, both sheets had similar appearances, with Figure 5a being the shielding film mixed with polymer and thermoformed and Figure 5b being the shielding sheet. The shielding film appeared darker in color as a result of the heat treatment during the pressing process. Additionally, the shielding film exhibited less flexibility compared to the shielding sheet, resembling a plate in terms of its strength and rigidity.

**Figure 6:**
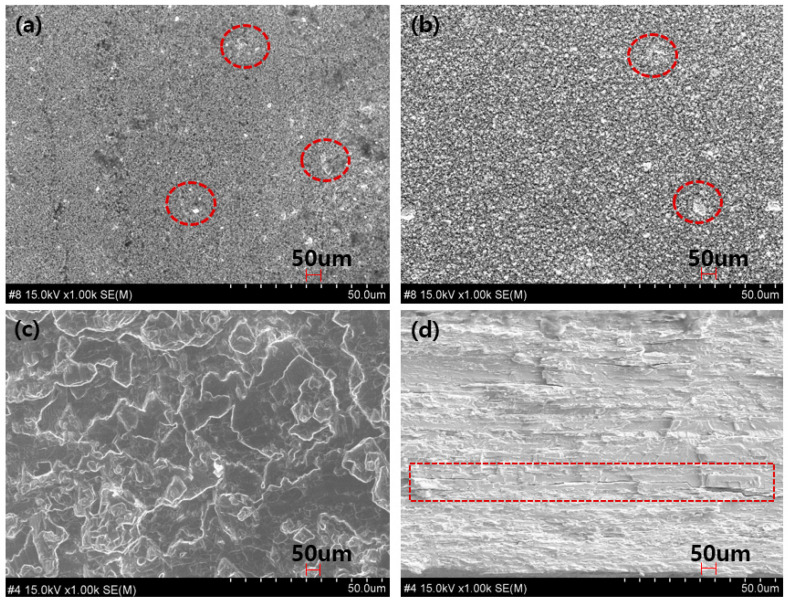
Enlarged view of the cross-section and surface of the shielding sheet produced using the calender process and the shielding body with the added compression process. Panel (**a**) is the surface image; (**b**) is the cross-sectional image, and the red circle is a sign that the polymer resin is agglomerated; (**c**) is the surface image of the shielding film imitating the laminated structure; and (**d**) is the cross-sectional image. The red square indicates the stacked form.

The cross-section and surface of the fabricated shielding film and shielding sheet were analyzed using an electron microscope. Firstly, the shielding sheet, commonly used as an eco-friendly shielding material, was examined, as shown in Figure 6. The surface images in Figure 6a and the cross-section in Figure 6b reveal clumping in the sheet manufactured through the calendering process by mixing tungsten and liquid polymer resin, indicating the lack of affinity between the polymer resin and tungsten. The improvement in the shielding performance of this structure could be attributed to the uniform dispersion of tungsten particles, indicating a well-configured area ratio distribution between the tungsten particles and polymer resin. At 85 wt% tungsten content, the dispersion of tungsten particles was possible, as shown in Figure 6a,b. However, it was found that during the process of mixing the liquid polymer resin, it was impossible to completely eliminate the phenomenon of polymer bonding or tungsten agglomeration. Therefore, the dispersion of tungsten particles is directly related to shielding performance. To overcome this, a method to minimize the gap between particles is needed.

The electron microscope image of the shielding film produced through the press compression process after molding through an injection molding machine is shown in Figure 6. Figure 6c is a surface image, and Figure 6d is a cross-sectional image. The results of the work can be checked by repeating the press compression of multiple layers. The red box in Figure 6d represents the layered structure, and it was confirmed that a similar layered structure could be obtained depending on the state of the injection solution. In this structure, the cooling rate is different for each layer, so the formed shielding film can lose strength over time, and the shielding film with a thickness of 0.3 mm has little flexibility. In this experiment, the shielding rate was evaluated to determine whether the laminated shielding-film structure helped improve radiation-shielding performance. The results of the shielding-rate experiment are shown in Table 1. Between the shielding sheet and the shielding film, the shielding film was superior. In particular, the shielding film was found to have a somewhat higher shielding effect against X-rays with high tube voltage than against X-rays with low tube voltage. Moreover, the laminated shielding film and the lead plate showed similar shielding rates for X-rays with low tube voltage. However, the results showed a clear difference in the shielding ratio in the X-ray area with high tube voltage.

The variation in the evaluation of the radiation-shielding rate of the shielding sheet with respect to the X-ray tube voltage is attributed to the agglomeration of liquid polymer and tungsten powder during the mixing process, resulting in regional differences in shielding performance, which showed better results at higher radiation energy intensities.

**Figure 7:**
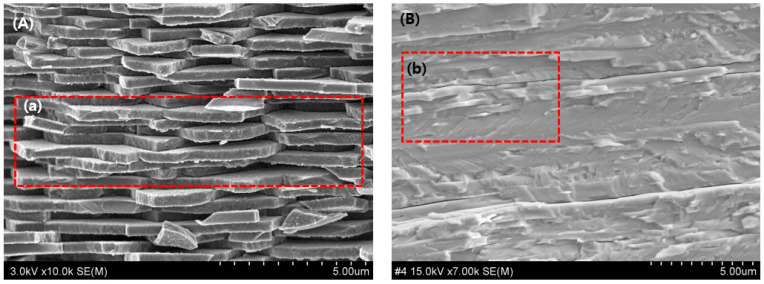
Comparison of the internal structure of the abalone shell (**A**) and the internal structure of the shield (**B**). The shape of (**a**) is a laminated structure, and the imitation pattern of (**b**) is a structure that reproduces the laminated structure through a press compression process.

Meanwhile, the shielding film exhibited a laminated structure as depicted in Figure 7. Figure 7A represents the horizontal structure of the inner layer of an abalone shell, while Figure 7B shows the laminated structure achieved through press compression fixation. Unlike the dispersion state of tungsten particles in the cross-sectional structure of the shielding sheet, the shielding film is composed of several layers without any gaps, as evident in Figure 7b. There is no space inside the laminated structure, and a few layers are formed between other layers because of the temperature difference during the bonding process, which results in a laminated structure. It can be confirmed that the actual inner layer of the abalone shell has a similar composition with air and protein layers intersecting, as depicted in Figure 7a.

## 4. Discussion

Key requirements for developing environmentally friendly shielding materials for medical radiation include achieving thin film conditions to reduce thickness and lightweight conditions to minimize shield weight [32]. However, these conditions often conflict during the manufacturing process. Increasing the radiation-shielding rate typically involves maximizing the incident radiation impact area and reducing strength, which can be challenging as it often necessitates increasing the thickness and content of the shielding material [33]. To address this, a novel approach focuses on the internal structure of the shielding body to enhance the shielding rate independently of thickness and content.

Even with identical content and thickness, shielding performance can be improved by manipulating the structural changes between shielding particles. This study found that the internal layered structure played a crucial role in reducing energy intensity by increasing interactions with incident radiation. To mimic the abalone shell’s inner layer structure, a new pattern structure was proposed, and the composite shielding material, prepared in the injection machine, was repeatedly manufactured in multiple layers through press compression in the mold. While manufacturing with an actual air layer could potentially enhance shielding performance, the thickness of the shielding film was kept as small as possible, considering the shielding-film requirements [34].

This technique draws inspiration from the internal structure of an abalone shell, primarily used for distributing loads in architectural applications. In this study, it serves to disperse the intensity of incoming energy. As the thickness and laminated structure increase, the flexibility of the shielding film decreases, but the shielding effect increases. The shielding film primarily utilizes tungsten as the main component, with polyamide nylon resin acting as the polymer material, providing versatility during the manufacturing process [35]. Adjusting the shield’s thickness can protect against the desired energy intensities, given that medical radiation is artificial and generates fixed energy [36]. The proposed layered structure could potentially be used to develop a systematic radiation-defense model as energy attenuation occurs as a result of the scattering of incident photons. As evidenced by this study, the shielding rate of the laminated structure outperformed that of the sheet manufactured using tungsten.

Conventional thin-film sheet research usually involves an even distribution of tungsten particles [37]. The objective is to maintain a uniform spacing between these particles, encapsulated by a polymer in dispersed form. Our study seeks to address this limitation during the fabrication process. By reproducing a repeated structure with a layered structure, the radiation collision area between particles can be expanded. Introducing a heat-press compression process at 180 °C to 220 °C to create a laminated structure makes the shielding film less flexible than the sheet but improves shielding performance [38]. While the high production cost may pose a challenge, the use of tungsten, an environmentally friendly material, allows for the creation of safe shielding products that are harmless to the human body [39].

Recently, much research has been conducted on radiation-shielding films made of eco-friendly materials that can replace lead. Lead is a heavy metal that is very difficult to dispose of after use. To overcome environmental factors, various materials such as tungsten, barium sulfate, bismuth, and boron have been receiving attention; however, the mechanical strength and cost-effectiveness of the product are also crucial factors. Therefore, in this study, tungsten was used to secure the shielding performance and solve internal structural problems by controlling the thickness and weight. Radiation shielding in medical institutions can take the form of a variety of tools inside medical equipment and imaging rooms, and it is expected to ensure safe defense and user activity. Therefore, the layered shielding film presented in this paper is a good material that can influence thin films and lightweight materials, and various types of models can be proposed according to changes in composite materials [40]. In addition, compared with products made through the calender process using tungsten, sheets with a laminated structure are believed to have better shielding performance and to be an efficient replacement for lead. In particular, it can be the most reasonable process technology for developing shielding materials for each medical radiation energy area. Therefore, in future research, we hope to move ahead from the proportional relationship between thickness and shielding performance, and we expect changes in shielding performance according to changes in internal structure, that is, patterns.

## 5. Conclusions

The same shielding structure was implemented through an injection molding process by imitating the internal layered structure pattern of the abalone shell. A 0.3 mm shielding film was manufactured using 85 wt% tungsten powder and polymer resin composite material. In the high-energy region, the shielding rate was more than 7% higher than that of the shielding sheet produced by mixing with liquid polymer. Compared with that of a lead plate of the same thickness, the shielding ratio of the shielding film was approximately 16% lower at 120 kVp. Therefore, the structure of the shielding film, which is identical to the internal laminated structure of the abalone shell, is confirmed to play a sufficient role in attenuating the intensity of the near-death energy of incident medical radiation and to contribute to improving the shielding performance. It is believed that in the future, reducing the thickness and weight of the shield such that it ensures the mobility of medical personnel will be feasible by changing the internal structure of the shield manufacturing process, and a shielding performance equivalent to that of lead plates will be achieved if the content of the shielding material and density of the molding process are increased. Through this study, it was confirmed that the laminated structure, the internal structure of the abalone shell, is a good alternative that improves radiation-shielding performance.

## Figures and Tables

**Table 1:** Shielding performance evaluation according to the process.

Tube Voltage (kVp)	Mean of Exposure (μR)				Shielding Rate (%)		
	No Shield	Conventional Rolled Shield	Abalone-Shell-Type Shield	Lead Plate	Conventional Rolled Shield	Abalone-Shell-Type Shield	Lead Plate
40	105.74	3.9230	1.9562	1.8927	96.29	98.15	98.21
60	405.73	61.7521	36.0694	8.1552	84.78	91.11	97.99
80	899.06	220.3596	151.8512	31.1075	75.49	83.11	96.54
100	1523.64	426.3145	317.0695	66.4307	72.02	79.19	95.64
120	1817.91	537.1924	410.3023	119.6185	70.45	77.43	93.42

## Data Availability

All data generated or analyzed during this study are included in this published article.
